# *trans*-Tetra­aqua­bis­(isonicotinamide-κ*N* ^1^)zinc bis­(3-hy­droxy­benzoate) tetra­hydrate

**DOI:** 10.1107/S1600536813006466

**Published:** 2013-03-13

**Authors:** Ibrahim Göker Zaman, Nagihan Çaylak Delibaş, Hacali Necefoğlu, Tuncer Hökelek

**Affiliations:** a Department of Chemistry, Kafkas University, 36100 Kars, Turkey; b Department of Physics, Sakarya University, 54187 Esentepe, Sakarya, Turkey; c Department of Physics, Hacettepe University, 06800 Beytepe, Ankara, Turkey

## Abstract

The asymmetric unit of the title compound, [Zn(C_6_H_6_N_2_O)_2_(H_2_O)_4_](C_7_H_5_O_3_)_2_·4H_2_O, contains half of the complex cation with the Zn^II^ ion located on an inversion center, a 3-hy­droxy­benzoate counter-anion and two uncoordinating water mol­ecules. Four water O atoms in the equatorial plane around the Zn^II^ ion [Zn—O = 2.089 (2) and 2.128 (2) Å] form a slightly distorted square-planar arrangement and the distorted octa­hedral geometry is completed by the two N atoms [Zn—N = 2.117 (2) Å] from two isonicotinamide ligands. In the anion, the carboxyl­ate group is twisted from the attached benzene ring at 9.0 (2)°. In the crystal, a three-dimensional hydrogen-bonding network, formed by classical O—H⋯O and N—H⋯O and weak C—H⋯O hydrogen bonds, consolidates the crystal packing, which exhibits π–π stacking between the benzene and pyridine rings, with centroid–centroid distances of 3.458 (2) and 3.609 (2) Å. One of the two H atoms of each uncoordinating water mol­ecule is disordered over two orientations with an occupancy ratio of 0.60:0.40.

## Related literature

For related structures, see: Hökelek *et al.* (2009*a* ▶,*b* ▶,*c* ▶,*d* ▶,*e* ▶); Sertçelik *et al.* (2009**a* ▶,b* ▶). For isostructural Ni and Co complexes, see: Zaman *et al.* (2012**a* ▶,b* ▶).
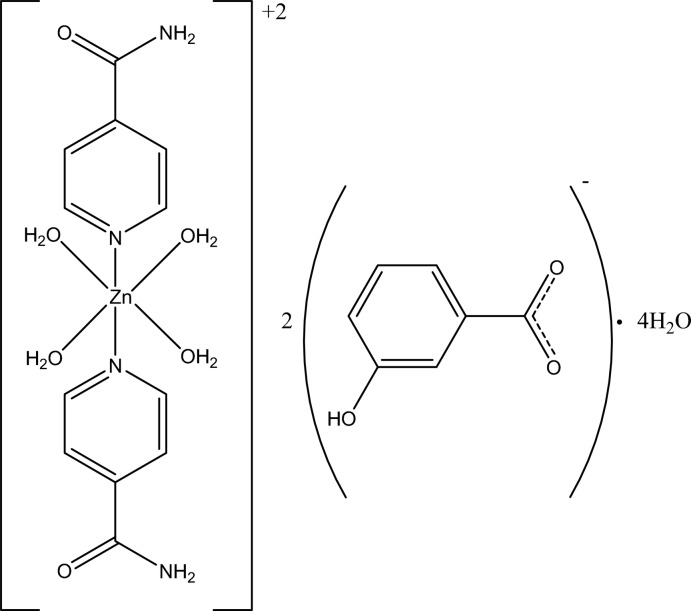


## Experimental

### 

#### Crystal data

[Zn(C_6_H_6_N_2_O)_2_(H_2_O)_4_](C_7_H_5_O_3_)_2_·4H_2_O
*M* *_r_* = 727.99
Monoclinic, 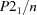

*a* = 6.7002 (2) Å
*b* = 17.0005 (4) Å
*c* = 13.6000 (3) Å
β = 99.993 (3)°
*V* = 1525.63 (7) Å^3^
*Z* = 2
Mo *K*α radiation
μ = 0.89 mm^−1^
*T* = 100 K
0.38 × 0.38 × 0.32 mm

#### Data collection

Bruker Kappa APEXII CCD area-detector diffractometer
Absorption correction: multi-scan (*SADABS*; Bruker, 2005 ▶) *T*_min_ = 0.720, *T*_max_ = 0.752
14180 measured reflections
3808 independent reflections
3497 reflections with *I* > 2σ(*I*)
*R*_int_ = 0.033

#### Refinement

*R*[*F* ^2^ > 2σ(*F* ^2^)] = 0.047
*wR*(*F* ^2^) = 0.114
*S* = 1.27
3808 reflections
264 parameters
H atoms treated by a mixture of independent and constrained refinement
Δρ_max_ = 1.41 e Å^−3^
Δρ_min_ = −0.47 e Å^−3^

### 

Data collection: *APEX2* (Bruker, 2007 ▶); cell refinement: *SAINT* (Bruker, 2007 ▶); data reduction: *SAINT*; program(s) used to solve structure: *SHELXS97* (Sheldrick, 2008 ▶); program(s) used to refine structure: *SHELXL97* (Sheldrick, 2008 ▶); molecular graphics: *ORTEP-3 for Windows* (Farrugia, 2012 ▶); software used to prepare material for publication: *WinGX* (Farrugia, 2012 ▶) and *PLATON* (Spek, 2009 ▶).

## Supplementary Material

Crystal structure: contains datablock(s) I, global. DOI: 10.1107/S1600536813006466/xu5683sup1.cif

Structure factors: contains datablock(s) I. DOI: 10.1107/S1600536813006466/xu5683Isup2.hkl

Additional supplementary materials:  crystallographic information; 3D view; checkCIF report

## Figures and Tables

**Table 1:** Hydrogen-bond geometry (Å, °)

*D*—H⋯*A*	*D*—H	H⋯*A*	*D*⋯*A*	*D*—H⋯*A*
O3—H3*A*⋯O8^i^	0.83 (5)	1.88 (5)	2.705 (3)	172 (5)
N2—H21⋯O7^ii^	0.83 (4)	2.24 (4)	3.017 (3)	157 (3)
N2—H22⋯O2^i^	0.82 (4)	2.21 (4)	3.016 (3)	172 (3)
O5—H51⋯O2^iii^	0.85 (5)	1.98 (5)	2.800 (3)	162 (4)
O5—H52⋯O3^ii^	0.76 (4)	1.97 (4)	2.719 (3)	170 (4)
O6—H61⋯O2^iv^	0.83 (5)	1.89 (5)	2.689 (3)	161 (5)
O6—H62⋯O4^v^	0.77 (5)	1.92 (5)	2.687 (3)	179 (5)
O7—H71⋯O1	0.85 (5)	1.91 (5)	2.761 (3)	178 (3)
O7—H72*A*⋯O8^vi^	0.76 (9)	2.08 (9)	2.814 (4)	163 (8)
O7—H72*B*⋯O7^vii^	0.78 (9)	2.03 (9)	2.783 (3)	160 (8)
O8—H81⋯O1	0.89 (5)	1.85 (5)	2.739 (3)	177 (4)
O8—H82*A*⋯O7^viii^	0.69 (8)	2.13 (8)	2.814 (4)	167 (6)
O8—H82*B*⋯O8^ix^	0.82 (9)	1.96 (9)	2.787 (3)	178 (6)
C11—H11⋯O7^ii^	0.93	2.54	3.455 (3)	168

Symmetry codes: (i) 
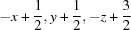
; (ii) 
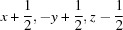
; (iii) 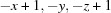; (iv) 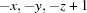; (v) 
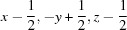
; (vi) 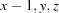; (vii) 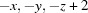; (viii) 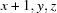; (ix) 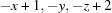.

**Fig. 1.**
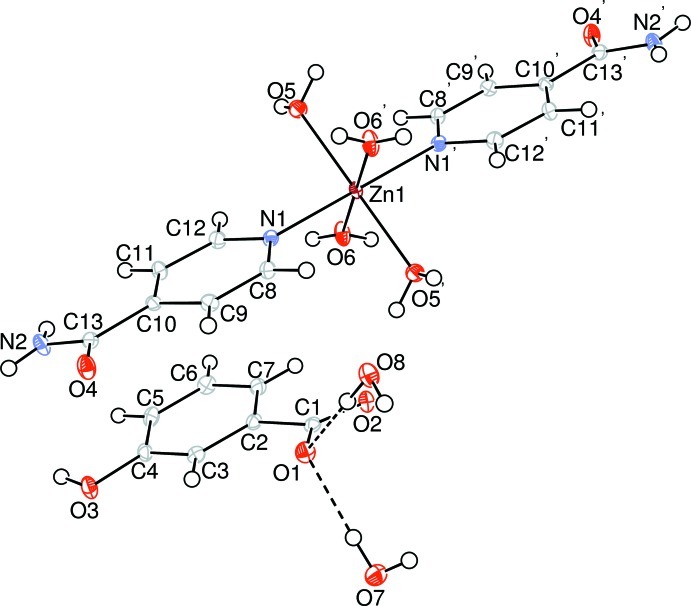
The molecular structure of the title molecule with the atom-numbering scheme. Displacement ellipsoids are drawn at the 50% probability level. Primed atoms are generated by the symmetry operator: (') - x, - y, - z. Only one of the disordered hydrogen atoms for each of the two uncoordinated water molecules is shown for clarity. Hydrogen bonds are shown as dashed lines.

## supplementary crystallographic information

### Comment

As a part of our ongoing investigation on transition metal complexes of nicotinamide (NA) and/or the nicotinic acid derivative *N*,*N*-diethylnicotinamide (DENA) (Hökelek *et al.*, 2009*a*; Hökelek *et al.*, 2009*b*; Hökelek *et al.*, 2009*c*; Hökelek *et al.*, 2009*d*; Hökelek *et al.*, 2009*e*; Sertçelik *et al.*, 2009**a*,b*), the title compound was synthesized and its crystal structure is reported herein.

The title compound (I) is isostructural with the related Ni (Zaman *et al.*, 2012*a*) and Co (Zaman *et al.*, 2012*b*) complexes. In (I) (Fig. 1), four O atoms (O5, O6, and the symmetry-related atoms, O5', O6') in the equatorial plane around the Zn atom form a slightly distorted square-planar arrangement, while the slightly distorted octahedral coordination is completed by the two pyridine N atoms (N1, N1') of the INA ligands at 2.117 (2) Å from the Zn atom in the axial positions (Fig. 1). The average Zn—O bond length is 2.108 (2) Å. The intramolecular O—H···O hydrogen bonds (Table 1)link the uncoordinated water molecules to the HB anion. The dihedral angle between the planar carboxylate group (O1/O2/C1) and the benzene ring A (C2—C7) is 9.0 (2)°, while that between rings A and B (N1/C8—C12) is 1.26 (8)°.

In the crystal structure, intermolecular O—H···O, N—H···O and weak C—H···O hydrogen bonds (Table 1) link the molecules into a three-dimensional network, in which they may be effective in the stabilization of the structure. π···π Contacts between the benzene and phenyl rings, *Cg*1—*Cg*2 and *Cg*2—*Cg*1^i^, [symmetry code: (i) 1 + *x*, *y*, *z*, where *Cg*1 and *Cg*2 are centroids of the rings A (C2—C7) and B (N1/C8—C12), respectively] may further stabilize the structure, with centroid-centroid distances of 3.609 (2) and 3.458 (2) Å, respectively.

### Experimental

The title compound was prepared by the reaction of ZnSO_4_.H_2_O (0.89 g, 5 mmol) in H_2_O (100 ml) and INA (1.220 g, 10 mmol) in H_2_O (50 ml) with sodium 3-hydroxybenzoate (1.601 g, 10 mmol) in H_2_O (100 ml). The mixture was filtered and set aside to crystallize at ambient temperature for four weeks, giving colorless single crystals.

### Refinement

Atoms H51, H52, H61, H62, H71, H72, H81 and H82 (for H_2_O), H21 and H22 (for NH_2_) and H3A (for OH) were located in a difference Fourier map and refined isotropically. The C-bound H-atoms were positioned geometrically with C—H = 0.93 Å, for aromatic H-atoms, and constrained to ride on their parent atoms, with *U*_iso_(H) = 1.2*U*_eq_(C). During the refinement process the disordered H72A, H82A and H72B, H82B atoms were refined with occupancies ratios of 0.60:0.40. The highest residual electron density was found 1.38 Å from O6 and the deepest hole 0.83 Å from H61.

### Crystal data

**Table:**

[Zn(C_6_H_6_N_2_O)_2_(H_2_O)_4_](C_7_H_5_O_3_)_2_·4H_2_O	*F*(000) = 760
*M**_r_* = 727.99	*D*_x_ = 1.585 Mg m^−^^3^
Monoclinic, *P*2_1_/*n*	Mo *K*α radiation, λ = 0.71073 Å
Hall symbol: -P 2yn	Cell parameters from 7045 reflections
*a* = 6.7002 (2) Å	θ = 2.4–28.4°
*b* = 17.0005 (4) Å	µ = 0.89 mm^−^^1^
*c* = 13.6000 (3) Å	*T* = 100 K
β = 99.993 (3)°	Block, colorless
*V* = 1525.63 (7) Å^3^	0.38 × 0.38 × 0.32 mm
*Z* = 2	

### Data collection

**Table:**

Bruker Kappa APEXII CCD area-detector diffractometer	3808 independent reflections
Radiation source: fine-focus sealed tube	3497 reflections with *I* > 2σ(*I*)
Graphite monochromator	*R*_int_ = 0.033
φ and ω scans	θ_max_ = 28.5°, θ_min_ = 2.4°
Absorption correction: multi-scan (*SADABS*; Bruker, 2005)	*h* = −8→8
*T*_min_ = 0.720, *T*_max_ = 0.752	*k* = −22→22
14180 measured reflections	*l* = −18→18

### Refinement

**Table:**

Refinement on *F*^2^	Primary atom site location: structure-invariant direct methods
Least-squares matrix: full	Secondary atom site location: difference Fourier map
*R*[*F*^2^ > 2σ(*F*^2^)] = 0.047	Hydrogen site location: inferred from neighbouring sites
*wR*(*F*^2^) = 0.114	H atoms treated by a mixture of independent and constrained refinement
*S* = 1.27	*w* = 1/[σ^2^(*F*_o_^2^) + (0.0244*P*)^2^ + 3.4855*P*] where *P* = (*F*_o_^2^ + 2*F*_c_^2^)/3
3808 reflections	(Δ/σ)_max_ < 0.001
264 parameters	Δρ_max_ = 1.41 e Å^−^^3^
0 restraints	Δρ_min_ = −0.47 e Å^−^^3^

### Special details

**Table:**

Geometry. All e.s.d.'s (except the e.s.d. in the dihedral angle between two l.s. planes) are estimated using the full covariance matrix. The cell e.s.d.'s are taken into account individually in the estimation of e.s.d.'s in distances, angles and torsion angles; correlations between e.s.d.'s in cell parameters are only used when they are defined by crystal symmetry. An approximate (isotropic) treatment of cell e.s.d.'s is used for estimating e.s.d.'s involving l.s. planes.
Refinement. Refinement of *F*^2^ against ALL reflections. The weighted *R*-factor *wR* and goodness of fit *S* are based on *F*^2^, conventional *R*-factors *R* are based on *F*, with *F* set to zero for negative *F*^2^. The threshold expression of *F*^2^ > σ(*F*^2^) is used only for calculating *R*-factors(gt) *etc*. and is not relevant to the choice of reflections for refinement. *R*-factors based on *F*^2^ are statistically about twice as large as those based on *F*, and *R*- factors based on ALL data will be even larger.

### Fractional atomic coordinates and isotropic or equivalent isotropic displacement parameters (Å^2^)

**Table:**

	*x*	*y*	*z*	*U*_iso_*/*U*_eq_	Occ. (<1)
Zn1	0.5000	0.0000	0.5000	0.00963 (12)	
O1	0.1790 (3)	0.11717 (11)	0.83803 (14)	0.0153 (4)	
O2	0.0781 (3)	0.04165 (11)	0.70518 (14)	0.0141 (4)	
O3	0.0140 (3)	0.39187 (11)	0.71927 (15)	0.0172 (4)	
H3A	0.004 (7)	0.428 (3)	0.678 (4)	0.051 (14)*	
O4	0.6024 (3)	0.33081 (11)	0.81904 (14)	0.0168 (4)	
O5	0.6799 (3)	0.04833 (12)	0.40047 (15)	0.0144 (4)	
H51	0.763 (7)	0.017 (3)	0.381 (3)	0.041 (12)*	
H52	0.636 (6)	0.070 (2)	0.353 (3)	0.028 (11)*	
O6	0.2262 (3)	0.03391 (12)	0.41227 (15)	0.0166 (4)	
H61	0.140 (8)	0.002 (3)	0.385 (4)	0.052 (14)*	
H62	0.192 (6)	0.073 (3)	0.386 (3)	0.037 (12)*	
O7	−0.0930 (4)	0.06755 (13)	0.95598 (17)	0.0201 (4)	
H71	−0.009 (7)	0.084 (3)	0.920 (3)	0.040 (12)*	
H72A	−0.201 (14)	0.059 (5)	0.930 (6)	0.05 (2)*	0.60
H72B	−0.05 (2)	0.026 (8)	0.967 (9)	0.059*	0.40
O8	0.5012 (4)	0.01950 (12)	0.90057 (16)	0.0171 (4)	
H81	0.398 (7)	0.052 (3)	0.882 (3)	0.045 (13)*	
H82A	0.596 (12)	0.037 (4)	0.910 (5)	0.03 (2)*	0.60
H82B	0.497 (16)	0.008 (6)	0.959 (7)	0.030*	0.40
N1	0.5135 (3)	0.10959 (12)	0.57473 (16)	0.0104 (4)	
N2	0.4845 (4)	0.39638 (14)	0.67657 (18)	0.0148 (5)	
H21	0.440 (6)	0.395 (2)	0.616 (3)	0.037 (11)*	
H22	0.474 (5)	0.438 (2)	0.705 (3)	0.019 (9)*	
C1	0.1097 (4)	0.10822 (15)	0.74642 (19)	0.0111 (5)	
C2	0.0586 (4)	0.18077 (15)	0.68340 (19)	0.0109 (5)	
C3	0.0647 (4)	0.25421 (15)	0.72947 (19)	0.0117 (5)	
H3	0.1021	0.2584	0.7984	0.014*	
C4	0.0147 (4)	0.32091 (15)	0.67183 (19)	0.0124 (5)	
C5	−0.0367 (4)	0.31508 (15)	0.5682 (2)	0.0144 (5)	
H5	−0.0688	0.3600	0.5298	0.017*	
C6	−0.0395 (4)	0.24190 (16)	0.5227 (2)	0.0149 (5)	
H6	−0.0723	0.2380	0.4536	0.018*	
C7	0.0063 (4)	0.17457 (15)	0.57989 (19)	0.0132 (5)	
H7	0.0022	0.1255	0.5493	0.016*	
C8	0.5621 (4)	0.11492 (15)	0.67488 (19)	0.0120 (5)	
H8	0.5891	0.0689	0.7118	0.014*	
C9	0.5736 (4)	0.18572 (15)	0.72495 (19)	0.0120 (5)	
H9	0.6091	0.1871	0.7941	0.014*	
C10	0.5317 (4)	0.25512 (14)	0.67106 (19)	0.0101 (5)	
C11	0.4793 (4)	0.24979 (15)	0.56786 (19)	0.0122 (5)	
H11	0.4491	0.2948	0.5293	0.015*	
C12	0.4728 (4)	0.17650 (15)	0.52330 (19)	0.0127 (5)	
H12	0.4385	0.1736	0.4541	0.015*	
C13	0.5426 (4)	0.33143 (15)	0.72775 (19)	0.0118 (5)	

### Atomic displacement parameters (Å^2^)

**Table:**

	*U*^11^	*U*^22^	*U*^33^	*U*^12^	*U*^13^	*U*^23^
Zn1	0.0128 (2)	0.00708 (19)	0.00863 (19)	0.00011 (15)	0.00070 (14)	−0.00048 (15)
O1	0.0184 (10)	0.0151 (9)	0.0118 (9)	0.0005 (7)	0.0012 (7)	0.0016 (7)
O2	0.0166 (9)	0.0098 (8)	0.0150 (9)	−0.0010 (7)	0.0003 (7)	−0.0002 (7)
O3	0.0250 (11)	0.0099 (9)	0.0162 (9)	0.0003 (8)	0.0025 (8)	−0.0012 (7)
O4	0.0261 (11)	0.0121 (9)	0.0112 (9)	−0.0017 (8)	0.0006 (8)	−0.0013 (7)
O5	0.0175 (10)	0.0138 (9)	0.0125 (9)	0.0015 (8)	0.0048 (8)	0.0016 (7)
O6	0.0172 (10)	0.0104 (9)	0.0187 (10)	0.0010 (8)	−0.0061 (8)	0.0019 (8)
O7	0.0234 (12)	0.0196 (10)	0.0189 (10)	−0.0029 (9)	0.0078 (9)	−0.0003 (8)
O8	0.0185 (11)	0.0133 (9)	0.0183 (10)	0.0019 (8)	0.0000 (8)	−0.0001 (8)
N1	0.0094 (10)	0.0106 (10)	0.0110 (10)	−0.0018 (8)	0.0010 (8)	−0.0011 (8)
N2	0.0215 (12)	0.0100 (10)	0.0124 (11)	0.0012 (9)	0.0015 (9)	−0.0027 (8)
C1	0.0079 (11)	0.0122 (11)	0.0135 (12)	−0.0005 (9)	0.0028 (9)	0.0007 (9)
C2	0.0106 (11)	0.0105 (11)	0.0117 (11)	0.0000 (9)	0.0023 (9)	0.0023 (9)
C3	0.0095 (11)	0.0142 (12)	0.0107 (11)	−0.0013 (9)	0.0001 (9)	−0.0001 (9)
C4	0.0114 (11)	0.0098 (11)	0.0158 (12)	−0.0014 (9)	0.0016 (9)	−0.0018 (9)
C5	0.0150 (12)	0.0123 (12)	0.0152 (12)	−0.0013 (10)	0.0008 (10)	0.0038 (9)
C6	0.0162 (13)	0.0169 (13)	0.0110 (12)	−0.0017 (10)	0.0008 (9)	0.0004 (9)
C7	0.0144 (12)	0.0124 (11)	0.0130 (12)	−0.0016 (9)	0.0030 (9)	−0.0012 (9)
C8	0.0131 (12)	0.0101 (11)	0.0129 (12)	0.0004 (9)	0.0025 (9)	0.0014 (9)
C9	0.0127 (12)	0.0130 (12)	0.0102 (11)	0.0005 (9)	0.0015 (9)	−0.0002 (9)
C10	0.0081 (11)	0.0103 (11)	0.0122 (11)	−0.0006 (9)	0.0021 (9)	−0.0021 (9)
C11	0.0141 (12)	0.0095 (11)	0.0126 (12)	0.0014 (9)	0.0010 (9)	0.0009 (9)
C13	0.0117 (12)	0.0110 (11)	0.0131 (12)	−0.0018 (9)	0.0034 (9)	−0.0020 (9)
C12	0.0149 (12)	0.0125 (12)	0.0105 (11)	−0.0008 (9)	0.0020 (9)	−0.0009 (9)

### Geometric parameters (Å, º)

**Table:**

Zn1—O5	2.1276 (19)	N2—C13	1.327 (3)
Zn1—O5^i^	2.1276 (19)	N2—H21	0.83 (4)
Zn1—O6	2.089 (2)	N2—H22	0.81 (4)
Zn1—O6^i^	2.089 (2)	C2—C1	1.507 (3)
Zn1—N1	2.117 (2)	C2—C3	1.394 (3)
Zn1—N1^i^	2.117 (2)	C2—C7	1.394 (3)
O1—C1	1.261 (3)	C3—H3	0.9300
O2—C1	1.264 (3)	C4—C3	1.386 (4)
O3—C4	1.369 (3)	C5—C4	1.395 (4)
O3—H3A	0.83 (5)	C5—C6	1.388 (4)
O4—C13	1.237 (3)	C5—H5	0.9300
O5—H51	0.85 (5)	C6—H6	0.9300
O5—H52	0.76 (4)	C7—C6	1.388 (4)
O6—H61	0.83 (5)	C7—H7	0.9300
O6—H62	0.77 (5)	C8—C9	1.379 (3)
O7—H71	0.85 (5)	C8—H8	0.9300
O7—H72A	0.76 (9)	C9—C10	1.392 (3)
O7—H72B	0.78 (13)	C9—H9	0.9300
O8—H81	0.88 (5)	C10—C11	1.389 (3)
O8—H82A	0.69 (8)	C10—C13	1.504 (3)
O8—H82B	0.82 (9)	C11—C12	1.383 (3)
N1—C8	1.347 (3)	C11—H11	0.9300
N1—C12	1.338 (3)	C12—H12	0.9300
			
O5—Zn1—O5^i^	180.0	C3—C2—C1	119.4 (2)
O6—Zn1—O5	93.93 (8)	C3—C2—C7	120.3 (2)
O6^i^—Zn1—O5	86.07 (8)	C7—C2—C1	120.3 (2)
O6—Zn1—O5^i^	86.07 (8)	C2—C3—H3	120.2
O6^i^—Zn1—O5^i^	93.93 (8)	C4—C3—C2	119.5 (2)
O6—Zn1—O6^i^	180.00 (10)	C4—C3—H3	120.2
O6—Zn1—N1	89.51 (8)	O3—C4—C3	118.4 (2)
O6^i^—Zn1—N1	90.49 (8)	O3—C4—C5	121.2 (2)
O6—Zn1—N1^i^	90.49 (8)	C3—C4—C5	120.4 (2)
O6^i^—Zn1—N1^i^	89.51 (8)	C4—C5—H5	120.1
N1—Zn1—O5	89.07 (8)	C6—C5—C4	119.7 (2)
N1^i^—Zn1—O5	90.93 (8)	C6—C5—H5	120.1
N1—Zn1—O5^i^	90.93 (8)	C5—C6—H6	119.8
N1^i^—Zn1—O5^i^	89.07 (8)	C7—C6—C5	120.3 (2)
N1—Zn1—N1^i^	180.00 (5)	C7—C6—H6	119.8
C4—O3—H3A	110 (3)	C2—C7—H7	120.1
Zn1—O5—H51	116 (3)	C6—C7—C2	119.7 (2)
Zn1—O5—H52	123 (3)	C6—C7—H7	120.1
H52—O5—H51	102 (4)	N1—C8—C9	122.7 (2)
Zn1—O6—H61	123 (3)	N1—C8—H8	118.6
Zn1—O6—H62	131 (3)	C9—C8—H8	118.6
H61—O6—H62	103 (4)	C8—C9—C10	119.4 (2)
H71—O7—H72B	96 (10)	C8—C9—H9	120.3
H72A—O7—H71	118 (7)	C10—C9—H9	120.3
H72A—O7—H72B	104 (10)	C9—C10—C13	118.3 (2)
H81—O8—H82A	116 (6)	C11—C10—C9	118.0 (2)
H81—O8—H82B	106 (8)	C11—C10—C13	123.7 (2)
H82A—O8—H82B	96 (9)	C10—C11—H11	120.5
C8—N1—Zn1	121.81 (17)	C12—C11—C10	119.0 (2)
C12—N1—Zn1	120.62 (17)	C12—C11—H11	120.5
C12—N1—C8	117.6 (2)	N1—C12—C11	123.3 (2)
C13—N2—H21	122 (3)	N1—C12—H12	118.4
C13—N2—H22	121 (2)	C11—C12—H12	118.4
H22—N2—H21	117 (4)	O4—C13—N2	123.2 (2)
O1—C1—O2	123.4 (2)	O4—C13—C10	119.1 (2)
O1—C1—C2	118.1 (2)	N2—C13—C10	117.7 (2)
O2—C1—C2	118.4 (2)		
			
O5—Zn1—N1—C8	−131.4 (2)	C1—C2—C7—C6	−179.7 (2)
O5^i^—Zn1—N1—C8	48.6 (2)	C3—C2—C7—C6	0.1 (4)
O5—Zn1—N1—C12	48.9 (2)	O3—C4—C3—C2	177.4 (2)
O5^i^—Zn1—N1—C12	−131.1 (2)	C5—C4—C3—C2	−1.6 (4)
O6—Zn1—N1—C8	134.7 (2)	C6—C5—C4—O3	−178.3 (2)
O6^i^—Zn1—N1—C8	−45.3 (2)	C6—C5—C4—C3	0.6 (4)
O6—Zn1—N1—C12	−45.1 (2)	C4—C5—C6—C7	0.7 (4)
O6^i^—Zn1—N1—C12	134.9 (2)	C2—C7—C6—C5	−1.0 (4)
Zn1—N1—C8—C9	179.39 (19)	N1—C8—C9—C10	0.7 (4)
C12—N1—C8—C9	−0.8 (4)	C8—C9—C10—C11	0.1 (4)
Zn1—N1—C12—C11	−179.9 (2)	C8—C9—C10—C13	179.2 (2)
C8—N1—C12—C11	0.3 (4)	C9—C10—C11—C12	−0.6 (4)
C3—C2—C1—O1	−8.0 (4)	C13—C10—C11—C12	−179.6 (2)
C3—C2—C1—O2	170.9 (2)	C9—C10—C13—O4	5.2 (4)
C7—C2—C1—O1	171.8 (2)	C9—C10—C13—N2	−174.0 (2)
C7—C2—C1—O2	−9.3 (4)	C11—C10—C13—O4	−175.7 (2)
C1—C2—C3—C4	−179.0 (2)	C11—C10—C13—N2	5.1 (4)
C7—C2—C3—C4	1.2 (4)	C10—C11—C12—N1	0.4 (4)

Symmetry code: (i) −*x*+1, −*y*, −*z*+1.

### Hydrogen-bond geometry (Å, º)

**Table:**

*D*—H···*A*	*D*—H	H···*A*	*D*···*A*	*D*—H···*A*
O3—H3*A*···O8^ii^	0.83 (5)	1.88 (5)	2.705 (3)	172 (5)
N2—H21···O7^iii^	0.83 (4)	2.24 (4)	3.017 (3)	157 (3)
N2—H22···O2^ii^	0.82 (4)	2.21 (4)	3.016 (3)	172 (3)
O5—H51···O2^i^	0.85 (5)	1.98 (5)	2.800 (3)	162 (4)
O5—H52···O3^iii^	0.76 (4)	1.97 (4)	2.719 (3)	170 (4)
O6—H61···O2^iv^	0.83 (5)	1.89 (5)	2.689 (3)	161 (5)
O6—H62···O4^v^	0.77 (5)	1.92 (5)	2.687 (3)	179 (5)
O7—H71···O1	0.85 (5)	1.91 (5)	2.761 (3)	178 (3)
O7—H72*A*···O8^vi^	0.76 (9)	2.08 (9)	2.814 (4)	163 (8)
O7—H72*B*···O7^vii^	0.78 (9)	2.03 (9)	2.783 (3)	160 (8)
O8—H81···O1	0.89 (5)	1.85 (5)	2.739 (3)	177 (4)
O8—H82*A*···O7^viii^	0.69 (8)	2.13 (8)	2.814 (4)	167 (6)
O8—H82*B*···O8^ix^	0.82 (9)	1.96 (9)	2.787 (3)	178 (6)
C11—H11···O7^iii^	0.93	2.54	3.455 (3)	168

Symmetry codes: (i) −*x*+1, −*y*, −*z*+1; (ii) −*x*+1/2, *y*+1/2, −*z*+3/2; (iii) *x*+1/2, −*y*+1/2, *z*−1/2; (iv) −*x*, −*y*, −*z*+1; (v) *x*−1/2, −*y*+1/2, *z*−1/2; (vi) *x*−1, *y*, *z*; (vii) −*x*, −*y*, −*z*+2; (viii) *x*+1, *y*, *z*; (ix) −*x*+1, −*y*, −*z*+2.
